#  Postelimination Status of Childhood Leprosy: Report from a Tertiary-Care Hospital in South India

**DOI:** 10.1155/2013/328673

**Published:** 2013-09-08

**Authors:** P. Chaitra, Ramesh Marne Bhat

**Affiliations:** Department of Dermatology, Venereology and Leprosy, Father Muller Medical College, Mangalore, Karnataka 575002, India

## Abstract

*Introduction*. Leprosy, a statistically “eliminated” disease from the globe, continues to linger around in its endemic countries including India. *Objective*. This study describes the epidemiological and clinicopathological pattern of the disease seen in children over a period of 8 years following its elimination in India. *Materials and Methods*. Medical records of all leprosy cases up to 14 years of age registered between April 2005 and March 2013 were retrospectively analyzed. Data were retrieved using a predesigned proforma and entered into the database system for analysis. *Results*. Child proportion of newly registered leprosy cases did not show a significant decline in the years following its elimination. The disease seemed to manifest frequently in older children with an insignificant gender predilection. More than half of child cases had a history of household contact. Paucibacillary leprosy dominated in them with a solitary skin lesion as the most frequent presentation. Although nerve thickening was seen in nearly half of these children, neuritis and lepra reactions were less common. Deformity at the time of diagnosis was noted in 13.89% of cases. Although smear positivity was not a common feature in children affected with leprosy, a good clinicohistopathological correlation was observed in those who underwent biopsy. *Conclusion*. Our study and reports from different parts of the country depict the unturned curves in the epidemiology of childhood leprosy which mirrors active transmission in the community, lacunae in diagnosis, and the need to strengthen contact screening activities in the pediatric population to sustain elimination.

## 1. Introduction

Leprosy, once a major global public health problem, is now considered eliminated (less than 1 case per 10,000 population) from most of its endemic countries by the World Health Organization (WHO) [1]. At a national level, India achieved this target in December 2005 [2]. However, India leads the list of countries reporting high figures of leprosy globally, with 1,34,752 new cases detected as on 31st of March 2013 [1, 3]. Although there is a decline in the prevalence and new case detection rate in the recent years, the curve of children acquiring leprosy remains unturned accounting for more than 10% of the total new case load [3]. This reflects an active circulation of *M. leprae* bacilli in Indian communities building endemicity. With this setting, the present study aims at describing the epidemiological and clinicopathological pattern of the disease occurring in the pediatric population in order to help strengthen control activities in the “postelimination era.”

## 2. Materials and Methods

This descriptive retrospective study was conducted at the Father Muller Medical College Hospital, Mangalore, which is a charitable tertiary-care teaching hospital in Southern India serving Dakshina Kannada and its neighboring districts in Karnataka as well as Kerala. 

Medical records of all leprosy cases up to the age of 14 years registered by self-reporting between April 2005 and March 2013 were retrospectively assessed year wise. All cases registered received WHO recommended fixed duration multidrug therapy (MDT) after being categorized as multibacillary (MB) or paucibacillary (PB) based on number of skin and nerve lesions along with smear status of the patient [4].

Patient data at the time of diagnosis were retrieved onto a predesigned proforma which concerned the following variables at the time of registration: age, sex, history of household contact, number of skin lesions, nerve involvement in the form of thickening and/or tenderness, clinical classification [4, 5], presence of lepra reaction, slit-skin smear status, and available histopathology correlates. Treatment dropouts and relapse cases during the study period were also noted.

The collected data were analyzed using Microsoft Office Excel 2007 in the construction of tables. Statistical analysis of data was done using Chi-square test and Fisher's exact test.

## 3. Results

Of the total 280 new leprosy cases registered in the institute between 2005 and 2013, 36 were child cases up to 14 years of age. The average child proportion over a period of 8 years in the postelimination phase was 12.86%. (Table 1).

The majority (75%) belonged to the age group of 11–14 years, followed by 19.44% and 5.56% in the 6–10 years and 0–5 years age group. The youngest diseased child was of 3 years. Male to female sex ratio was 1.25 : 1. (Table 2).

The majority (75%) of the children formed the paucibacillary group, making the remainder 25% multibacillary. History of a household contact of leprosy was present in a large number (58.33%) of affected children in both disease groups (Table 3).

A solitary skin lesion (SSL) either a hypopigmented or an erythematous patch with decreased sensation with or without thickened nerve was the most frequent manifestation (61.11%) of leprosy in children followed by 2–5 skin lesions and more than 5 skin lesions. Thickened nerves were palpable in 17 out of 36 cases (47.22%) (Table 4).

Tuberculoid was the commonest clinical type (50%) followed by borderline tuberculoid (38.89%), indeterminate (5.56%), and borderline lepromatous (2.78%) types. No case of childhood pure neural leprosy was registered during this period. Histoid type was seen in a 14-year-old boy (2.78%) (Table 5).

Lepra reaction at the time of diagnosis was seen in 2 cases (5.56%) with a borderline tuberculoid patient presenting with type 1 reaction and neuritis and a borderline lepromatous case presenting with erythema nodosum leprosum (ENL).

Five cases (13.89%) had deformity at the time of diagnosis of which one case (2.78%) had visible deformity in the form of foot drop. No case had eye involvement.

Slit skin smears were done from 4 sites (ear lobule, forehead, chin, and buttock/thigh). A positive smear for acid-fast bacilli was found in 3 cases (8.33%) (Table 6).

Of the available biopsy records (27/36 cases), histopathology was conclusive of leprosy in 100% of cases. Tuberculoid (TT) type was the most common histological diagnosis (50%). A clinicohistopathological correlation was observed in 23 out of 27 cases (85.16%).

Out of the 36 child cases that commenced WHO-MDT, 32 completed the fixed duration treatment (88.89%), while 4 defaulted (11.11%), and 1 relapsed (2.78%). The 6-year-old boy who relapsed into multibacillary disease was released from PB-MDT a year ago. History of leprosy was present in his mother.

## 4. Discussion

The proportion of new child cases of leprosy in this part of the country remains high (more than 10% of new case load) and does not show a statistically significant decreasing trend over the last 8 years following elimination (*P* = 0.955).

Majority of pediatric cases of leprosy in our study belonged to the older age group that is above 11 years. Previous studies also reported a lesser occurrence in children less than 5 years [6–10]. A relatively long incubation period of leprosy may be one of the causes, and the chances of misdiagnosing indeterminate skin patches as pityriasis alba and tinea versicolor in the initial stages may also lead to delayed detection in these cases. However, leprosy can present in infancy as early as 3 weeks [11].

An insignificant male preponderance was seen in our study (*P* = 0.505). However, boys were considerably more in the other studies probably owing to their greater activity and increased opportunities for contact and neglect of female child in the study area [7, 8].

The proportion of contacts with leprosy is strikingly high in our study in concordance with other studies [8]. However, the type of disease (multibacillary or paucibacillary) in children exposed to leprosy contacts did not significantly differ from those unexposed children who developed the disease (*P* = 0.705). All the positive contacts were intrafamilial, and no extrafamilial contact history was available which may be due to stigmatic lack of disclosure of the disease in the neighborhood, if any. The risk of a person developing leprosy is four times higher when there is a neighborhood contact and up to 9 times higher when the contact is household [12]. This emphasizes the need for periodic screening of leprosy contacts specially the children in the family.

Single skin patch was the commonest symptom or sign of leprosy in children, which is similar to the observation of previous coworkers [6, 13]. A suspicion of a possibility of leprosy should arise in any child presenting with skin patches even if sensation is intact, and such cases should be observed for early detection.

Paucibacillary disease dominated in children in contrast to adults [7, 8, 14]. Some studies had higher number of multibacillary cases where the frequency of finding thickened nerves was high differentiating them into multibacillary group [6]. This stresses on a thorough examination of cutaneous nerves at the time of diagnosis to avoid undertreatment [15].

Smear positive leprosy is uncommon in childhood as witnessed in our report. However, a good number of bacillary cases are observed in children as well, mostly reported from endemic Northern India [7, 10].

Clinicohistopathological accordance in the studied biopsies was high maybe due to the higher number of determinate forms, although choice of the biopsy site adds to it [6]. Nonspecific histological features may be seen commonly in children owing to the still developing immune system in them [16].

Incidence of neuritis and reactions in children were low in our study in comparison with Jain et al. probably due to the inclusion of data recorded only at the time of registration [8]. Prompt and judicious steroid therapy should be instituted in such cases to avoid development of further neurological damage.

Deformity in children is an unfortunate tragedy. Factors that may contribute to deformities in children are the older age, multiple skin and nerve lesions, multibacillary disease, presence of reaction, smear positivity, and delayed diagnosis [17]. High occurrence of deformities (13.89%) and a case showing visible deformity at the time of diagnosis reflects the lacunae of the system in early case detection at the field level and referral services.

## 5. Conclusion

Leprosy continues to be a communicable disease of concern in the postelimination era.

Our study and reports from different parts of the country depict the unturned curves in the epidemiology of childhood leprosy in its endemic pockets which mirrors active transmission and delayed diagnosis in this age group. This alarms the need to strengthen contact screening, early case detection, and referral activities in the pediatric population to sustain elimination.

## Figures and Tables

**Table 1:** Year-wise proportion of child cases from 2005 to 2013.

Year	Total no. of new cases of leprosy	No. of child cases ≤ 14 yrs. of age	Child proportion (%)
April 2005–March 2006	29	3	10.35
April 2006–March 2007	36	5	13.89
April 2007–March 2008	27	4	14.82
April 2008–March 2009	34	6	17.63
April 2009–March 2010	39	3	7.69
April 2010–March 2011	26	4	15.39
April 2011–March 2012	56	7	12.5
April 2012–March 2013	33	4	12.12

Total	280	36	12.86

**Table 2:** Age and sex distribution.

Age (yrs.)	Total no. of cases (%)	Male			Female		
		PB	MB	Total (%)	PB	MB	Total (%)
0–5	2 (5.56)	1	0	1	1	0	1
6–10	7 (19.44)	5	0	5	2	0	2
11–14	27 (75)	8	6	14	10	3	13

Total	*N* = 36	14	6	20 (55.56)	13	3	16 (44.44)

**Table 3:** History of household contacts of leprosy and type of disease.

Type of leprosy	Household contact/s (*n* = 36)	
	Present	Absent
PB	15	12
MB	6	3

Total no. (%)	21 (58.33)	15 (41.67)

**Table 4:** Number of skin lesions and thickened nerves.

Age (yrs.)	No. of skin lesions (*n* = 36)			Thickened nerves (*n* = 36)
	SSL	2–5	>5	
0–5	1	1	0	0
6–10	5	2	0	1
11–14	16	6	5	16

Total (%)	22 (61.11)	9 (25)	5 (13.89)	17 (47.22)

**Table 5:** Clinical spectrum of childhood leprosy.

Age (yrs.)	TT*	BT*	BB*	BL*	LL*	I*	Histoid
0–5	1	1	0	0	0	0	0
6–10	5	2	0	0	0	0	0
11–14	12	11	0	1	0	2	1

Total (%)	18 (50)	14 (38.89)	0	1 (2.78)	0	2 (5.56)	1 (5.56)

*TT: tuberculoid, BT: borderline tuberculoid, BB: borderline borderline, BL: borderline lepromatous, LL: lepromatous, and I: indeterminate.

**Table 6:** Slit skin smear for acid-fast bacilli.

Clinical type (*n* = 36)	Smear status	
	Positive	Negative
TT	0	18
BT	1	13
BL	1	0
I	0	2
Histoid	1	0

Total (%)	3 (8.33)	33 (91.67)
